# Co-infection With Leptospirosis During Acute Hepatitis B Serologic Recovery: A Case Report

**DOI:** 10.7759/cureus.114805

**Published:** 2026-08-19

**Authors:** Duy T Dao, Xuan Yen T Nguyen, Ai Phuong T Truong

**Affiliations:** 1 Department of Internal Medicine, Pham Ngoc Thach University of Medicine, Ho Chi Minh, VNM; 2 Department of Gastroenterology, People's Hospital 115, Ho Chi Minh, VNM

**Keywords:** acute hepatitis b, acute liver injury, co-infection, jaundice, leptospirosis

## Abstract

Leptospirosis is a zoonotic infection that can cause acute liver injury and mimic viral hepatitis. We report a 30-year-old woman presenting with myalgia, progressive jaundice, and markedly elevated aminotransferases, initially diagnosed with acute hepatitis B. She reported regular contact with her dog. Despite serologic recovery with hepatitis B surface antigen seroconversion, severe hyperbilirubinemia and coagulopathy were atypical for resolving hepatitis B. Further evaluation revealed positive *Leptospira* immunoglobulin M (IgM) serology, and leptospirosis was diagnosed using modified Faine's criteria. Treatment with meropenem resulted in rapid clinical and biochemical improvement, highlighting leptospirosis as an important hidden cause of acute liver injury.

## Introduction

Leptospirosis is a globally important zoonotic infection caused by pathogenic *Leptospira* species, with the greatest burden occurring in tropical and subtropical regions because the climate and poor hygienic conditions favor the pathogen's survival and distribution, particularly in Southeast Asia [1]. Reported disease incidence ranged from 0.10 to 975 annual cases per 100,000 population. The annual morbidity and mortality due to leptospirosis worldwide were estimated to be 14.77 cases per 100,000 population and 0.84 deaths per 100,000 population, respectively [1]. Rodents, especially rats, are the most important reservoirs. Leptospirosis can be transmitted through direct contact with urine, blood, or tissue from an infected animal or more commonly through exposure to contaminated environments. Because leptospires can survive in a humid environment for months, water is an important vehicle for transmission [2].

Acute liver injury is a common clinical presentation in gastroenterology. Leptospirosis is an underrecognized cause of acute liver injury and can closely mimic acute viral hepatitis with tender hepatomegaly, jaundice, and elevated aminotransferases [2]. The clinical spectrum ranges from a mild, self-limited febrile illness to a severe, life-threatening condition, with a mortality rate approaching to 10% in severe cases [3]. Diagnostic challenges arise when leptospirosis presents concurrently with viral hepatitis, as serologic findings may be misleading. Spontaneous seroconversion during acute hepatitis B may falsely reassure clinicians, despite ongoing liver injury from an alternative etiology.

We report a case of leptospirosis presenting during the serologic recovery phase of acute hepatitis B, resulting in a diagnostic delay. This case highlights the importance of maintaining a broad differential diagnosis for acute liver injury and recognizing leptospirosis as a potential hidden cause, especially in regions where the disease is endemic.

## Case presentation

A 30-year-old woman was admitted with a 10-day history of myalgia, predominantly involving the back and legs, and nausea. Over the following five days, she developed chills, progressive jaundice, and scleral icterus, along with dark yellow urine, without abdominal pain. At that time, she was diagnosed with acute hepatitis B and was treated with tenofovir alafenamide 25 mg daily for five days at a previous hospital; however, her condition failed to improve. She had no known past medical history. She was not taking any medications, herbal products, or dietary supplements and had not received the hepatitis B vaccine. There was no family history of viral hepatitis. The patient reported regular contact with her dogs and no exposure to contaminated water. She was not a smoker.

The hematologic and biochemical results are shown in Table 1 and Table 2, indicating changes over different time points.

**Table 1:** Serial liver biochemistry and coagulation parameters INR: international normalized ratio; AST: aspartate aminotransferase; ALT: alanine aminotransferase; ALP: alkaline phosphatase; GGT: gamma-glutamyl transferase; -: data not available

Parameter	Reference range	Day 5 (outside hospital)	Day 0 (admission)	Day 2	Day 28 (follow-up)
AST (U/L)	<31	1601	617.2	211	21.4
ALT (U/L)	<31	2103	1706	788.6	12
ALP (U/L)	35-104	227.7	119.5	-	-
GGT (U/L)	5-36	-	124.5	-	-
Total bilirubin (µmol/L)	<17.1	93.5	128.07	77.05	12.42
Direct bilirubin (µmol/L)	<5.1	65.1	81.17	42.95	4.1
INR	1.14-1.27	-	1.73	1.03	0.86

**Table 2:** Hematologic and serologic findings ANA: anti-nuclear antibody; anti-HAV: hepatitis A virus antibody; anti-HCV: hepatitis C virus antibody; anti-HEV: hepatitis E virus antibody; HBsAg: hepatitis B surface antigen; anti-HBs: hepatitis B surface antibody; anti-LKM: liver-kidney microsomal antibody; SMA: smooth muscle antibody; eGFR: estimated glomerular filtration rate; CKD-EPI: Chronic Kidney Disease Epidemiology Collaboration; anti-HBc IgM (S/CO): antibody to hepatitis B core antigen immunoglobulin M (sample-to-cutoff ratio); HDV RNA: hepatitis D virus ribonucleic acid; AMA: anti-mitochondrial antibodies; -: data not available

Parameter	Reference range	Day 5 (outside hospital)	Day 0 (admission)	Day 28 (follow-up)
Neutrophil count (×10^3^/µL)	2.5-7.0	5.42	2.8	2.9
Red blood cell count (×10^6^/µL)	4.0-5.4	4.78	4.11	3.94
Hemoglobin (g/dL)	12.2-15.4	14.3	12.6	12.8
Platelets (×10^3^/µL)	150-400	290	313	372
Creatinine (µmol/L)	44-88	-	45.6	46.2
eGFR (CKD-EPI) (ml/min/1.73 m^2^)	-	-	129.21	128
HBsAg	<1	Positive (7.19)	Negative	Negative
Anti-HBs (mIU/ml)	0-10	Positive (82.87)	-	Positive (32.89)
Anti-HBc IgM (S/CO)	0.0-1.0	Reactive (42.2)	-	-
HDV RNA	-	-	Target not detected	-
Anti-HCV	<1	-	Negative	-
Anti-HAV IgM	Negative	-	Negative	-
Anti-HEV IgM	Negative	-	Negative	-
*Leptospira* IgM (U/ml)	<15	-	Positive (40.5)	-
Anti-LKM-1 (U/ml)	<12	-	Negative	-
AMA (U/ml)	<12	-	Negative	-
ANA	Negative	-	Negative	-
SMA	Negative	-	Negative	-

Urinalysis showed no proteinuria or hematuria, with negative nitrites and leukocytes.

Abdominal ultrasound revealed decreased hepatic echogenicity without other abnormalities. Therefore, abdominal magnetic resonance imaging was performed to rule out biliary obstruction.

**Figure 1:**
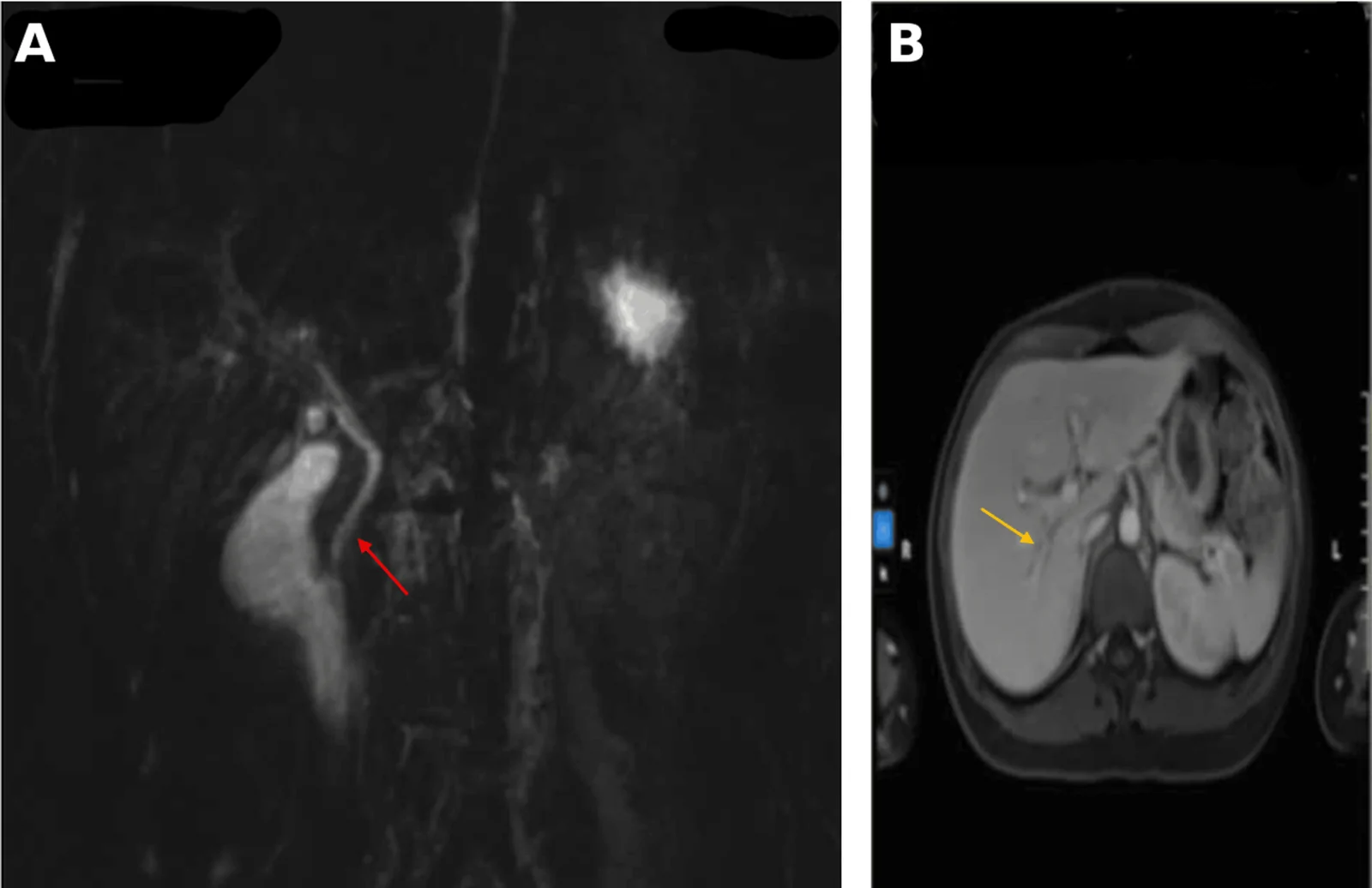
Abdominal magnetic resonance imaging findings (A) Magnetic resonance cholangiopancreatography showing a nondilated common bile duct (red arrow). (B) Axial magnetic resonance image demonstrating periportal edema (yellow arrow).

While magnetic resonance cholangiopancreatography showed a nondilated common bile duct, abdominal magnetic resonance imaging demonstrated periportal edema (Figure 1). Chest radiography revealed no abnormalities.

The patient was diagnosed with leptospirosis and additionally treated with meropenem 1 g three times daily for 14 days, along with fluid supplementation and vitamin K. Tenofovir alafenamide was stopped after the diagnosis of leptospirosis. Marked clinical improvement was observed within two days. She was discharged after two weeks and scheduled for a follow-up two weeks later.

## Discussion

This case highlights an unusual presentation of leptospirosis manifesting as acute liver injury. Hepatic involvement in leptospirosis can closely mimic acute viral hepatitis, presenting with jaundice, cholestasis, and elevated aminotransferases, as observed in our patient [2].

Acute hepatitis B is a self-limited infectious disease caused by hepatitis B virus, with spontaneous recovery occurring in more than 95% of immunocompetent patients with acute hepatitis B. Patients can present with fatigue, anorexia, nausea, jaundice, and markedly elevated aminotransferase levels. The diagnosis of acute hepatitis B is established by the presence of hepatitis B surface antigen (HBsAg) for less than six months or by the presence of immunoglobulin M (IgM) hepatitis B core antibody (anti-HBc) and hepatitis B surface antibody (anti-HBs) in the absence of HBsAg [4].

Leptospirosis is a globally important zoonotic infection caused by pathogenic *Leptospira* species, occurring predominantly in tropical and subtropical regions. Transmission occurs through direct contact with infected animal body fluids or exposure to contaminated water or soil. During the initial incubation period, leptospires can be isolated from the bloodstream. *Leptospira* can survive in the host by evading parts of the innate immune response such as complement-mediated killing and phagocytosis. Earlier studies have demonstrated an association between an exaggerated proinflammatory immune response and mortality. During the immune phase, the appearance of antibodies coincides with the disappearance of leptospires from the blood. Nevertheless, the bacteria can persist in various organs, including the liver, lungs, heart, brain, and kidneys [3]. Clinical severity ranges from mild illness characterized by fever, nausea, and vomiting to severe leptospirosis (Weil's syndrome), characterized by jaundice, hemorrhage, and acute kidney injury, with jaundice resulting primarily from cholestasis rather than hepatocellular necrosis [3,5]. Liver histopathology may demonstrate focal necrosis rather than widespread hepatocellular necrosis, foci of inflammation, and plugging of bile canaliculi. Experimental work showed the infiltration of *Leptospira* into the Disse space and migration between hepatocytes with detachment of the intercellular and disruption of bile canaliculi leading to bile leakage [3]. Because both diseases share overlapping clinical manifestations, distinguishing leptospirosis from acute hepatitis B based on clinical and biochemical findings can be challenging, particularly in atypical cases and endemic areas where both infections can coexist.

Diagnosis of leptospirosis remains challenging due to nonspecific clinical features and limitations of diagnostic testing. Faine's criteria and modified Faine's criteria, which integrate clinical, epidemiologic, and serologic findings, provide useful diagnostic tools, particularly in resource-limited settings [6,7]. Serologic tests may be negative early in the disease course, as antibodies typically develop after five days, whereas molecular techniques allow earlier detection but are not universally available [8].

Vietnam is located in a region with high leptospirosis endemicity; however, the disease remains underdiagnosed due to overlapping clinical features with other causes of acute liver injury and limited access to diagnostic testing [9]. In our patient, serologic testing demonstrated seroconversion from HBsAg to anti-HBs and the presence of IgM anti-HBc, suggesting recovery from acute hepatitis B [10]. Nevertheless, the persistence of severe hyperbilirubinemia and coagulopathy was atypical for resolving acute hepatitis B and prompted further evaluation. Subsequent testing revealed positive *Leptospira* IgM serology, supporting the diagnosis of leptospirosis based on modified Faine's criteria, with a total score of 29 points. Even though our case did not meet the classic criteria for Weil's syndrome, persistent hyperbilirubinemia and coagulation abnormalities were risk factors for severe disease in acute hepatitis. Despite common renal involvement in leptospirosis, its absence does not exclude the diagnosis [3].

Penicillin and doxycycline are first-line therapies for leptospirosis; meropenem has been reported to be effective in severe disease, especially when first-line antibiotics are unavailable or contraindicated [11,12]. Our patient showed rapid clinical and biochemical improvement following treatment with meropenem, which supports its potential role in severe leptospirosis. Moreover, the rapid clinical and biochemical response to antimicrobial therapy provided additional supportive evidence for the diagnosis of leptospirosis. However, the absence of confirmatory molecular testing or a microscopic agglutination test remained a limitation of our case.

To our knowledge, only a few cases of leptospirosis and hepatitis B co-infection have been reported. Previous reports described patients with Weil's syndrome and concurrent hepatitis B, whereas the nature of the hepatitis B virus infection was not clearly established [13,14]. In contrast, our patient had serologically confirmed acute hepatitis B in the recovery phase, while persistent biochemical abnormalities prompted further investigation and ultimately led to the diagnosis of leptospirosis, highlighting the importance of considering concurrent infections when the clinical course is atypical.

## Conclusions

This case highlights leptospirosis as an important differential diagnosis of acute liver injury, even in the absence of renal involvement. In patients with acute hepatitis B, persistent or disproportionate biochemical abnormalities despite serologic recovery should prompt evaluation for alternative or concurrent etiologies. Early recognition of leptospirosis and appropriate antimicrobial therapy may lead to rapid clinical improvement and favorable outcomes. However, the absence of confirmatory molecular testing or microscopic agglutination test limits the certainty of the causal relationship between meropenem treatment and clinical improvement.
